# “People say men don’t talk, well that’s bullshit”: A focus group study exploring challenges and opportunities for men’s mental health promotion

**DOI:** 10.1371/journal.pone.0261997

**Published:** 2022-01-21

**Authors:** Paul Sharp, Joan L. Bottorff, Simon Rice, John L. Oliffe, Nico Schulenkorf, Franco Impellizzeri, Cristina M. Caperchione

**Affiliations:** 1 School of Sport, Exercise and Rehabilitation, University of Technology Sydney, Sydney, New South Wales, Australia; 2 Institute for Healthy Living and Chronic Disease Prevention, University of British Columbia, Kelowna, British Columbia, Canada; 3 School of Nursing, University of British Columbia, Kelowna, British Columbia, Canada; 4 Orygen, Parkville, Melbourne, Victoria, Australia; 5 Centre for Youth Mental Health, University of Melbourne, Melbourne, Victoria, Australia; 6 School of Nursing, University of British Columbia, Vancouver, British Columbia, Canada; 7 Department of Nursing, University of Melbourne, Melbourne, Victoria, Australia; 8 Business School, University of Technology Sydney, Sydney, New South Wales, Australia; International Medical University, MALAYSIA

## Abstract

Men’s mental health promotion presents unique challenges including gender-related barriers and stigmas, which demand novel approaches to prevention, treatment, and management. The aim of this study was to explore men’s perceptions of mental health and preferences for mental health promotion. Seven focus groups (N = 59) were conducted in Sydney, Australia, including 5 groups of men (M = 50.65, SD = 13.75 years) and 2 groups of stakeholders who had frontline experience working with men (e.g., men’s groups, health clubs, mental health advocates). Data were analysed using thematic analysis and interpreted using a gender relations approach to explore connections between gender roles, relations and identities, and men’s mental health. Three overarching themes were identified; (1) *Roles*, *identities*, *and the conceptualisation and concealment of mental health challenges*, revealing challenges to mental health promotion related to perceptions of men’s restrictive emotionality and emotional awareness as well as difficulties with conceptualising the internalised experiences of mental health, (2) *Constraining social contexts of stigma and gender relations*, identifying how social context and the policing of gender roles often obscured opportunities for discussing mental health and help-seeking behaviour, (3) *Anchoring mental health promotion to acceptable lifestyle practices*, highlighting potential remedies included leveraging men’s social practices related to reciprocity, normalising mental health promotion relative to other behaviours, and embedding mental health promotion within acceptable masculine practices. Discussed are directions for men’s community-based mental health promotion and opportunities for how masculinities may be negotiated and expanded to embody mental health promoting values.

## Introduction

Consistent evidence has been found worldwide that suggests men are far less likely to seek help for mental health challenges, irrespective of age, nationality, or ethnic or racial background [1,2]. In Australia, men are less likely than women to access services for mental health disorders (28% vs. 41%), such as depression and anxiety, and they account for three-quarters of deaths from suicides [3,4]. Men experience mental health challenges diversely and the influence of gender, as a social determinant of health, has been well established [5,6]. For example, factors driving men’s resistance to seek help for psychological problems have been attributed to normative masculinities characterised by self-reliance, stoicism, and restrictive emotionality [5,7]. Additionally, gender-related barriers and stigmas to help-seeking contribute to many men’s poor mental health literacy and reticence towards mental health support and intervention [2,5,8]. Moreover, men are more likely to focus on physical symptoms (e.g., tiredness, irritability) and exhibit externalised behaviours (e.g., anger, substance use, risk-taking) that may not be recognised as by-products of mental health problems by friends, family or medical professionals [9,10]. As a result, mental health problems may be hidden or overlooked, and often go unrecognised or undiagnosed [11]. However, evidence also suggests that men will seek out and engage in health services when they are designed and delivered in ways that align with men’s preferences and interests [12], suggesting the need for gendered approaches to prevention and treatment. In this regard, there has been a growing interest in identifying strategies that engage men in mental health promotion and early intervention [13], including community-based mental health programs [14].

Mental health promotion is key to preventing men’s mental disorders, and by extension advancing the well-being of their families and friends. To date, efforts have focused on identifying and treating men’s mental health disorders, destigmatising mental illness, and norming men’s help-seeking behaviours [15]. Primary prevention and early intervention in men’s mental health is emergent with diverse community-based and e-health efforts augmenting traditional clinical services [16]. Men’s mental health promotion can work by reducing risk factors (e.g., stress, social-isolation, poor nutrition) and bolstering protective factors (e.g., emotional resilience, exercise, problem solving) to prevent mental ill-health and promote well-being [17]. As common risk and protective factors underpin many men’s mental health disorders (e.g., depression, anxiety, and substance-related disorders), effective mental health promotion efforts offer opportunities to prevent the onset, delay recurrence, and decrease the impact of a range of mental health disorders. Furthermore, mental health promotion programs can go beyond incidence reduction to exert positive effects on overall well-being and quality of life [18].

To better understand men’s mental health, we draw upon a gender relations approach to exploring masculinities and men’s mental health. Connell’s [19] early work on *hegemonic masculinity* has long been used to explain how, within specific locations, temporalities and context, characteristics and performativity’s are held up as the standard for idealised masculine behaviour. Hegemonic masculinity comprises dominant norms (characteristics and patriarchal power) to which men (and women) diversely align and/or are complicit in sustaining. Even though many men do not conform to normative masculinities, they may be complicit with, and even gain benefit from, the power relations involved. Whereby, other men (and women) may be marginalised (i.e., lacking the characteristics to conform to hegemonic masculinities) or subordinate (i.e., non-conforming, expressing qualities opposite to hegemonic masculinities) [7]. These concepts provide an overarching framework for understanding how gender is produced and reproduced through everyday social practices, arising from the complex interactions of agency, gender and socio-cultural influences [7]. Contemporary developments have recognised and explored the plurality of masculinities [20] as well as the influence that masculinities have on men’s health and health-related behaviours [21].

The relationship between masculinities and men’s mental health is diverse with variations on normative patterns including men’s stoicism and resistance to help-seeking. Normative masculinities often run counter to acknowledging and experiencing mental health problems. For example, expressing emotion such as sadness or crying may reduce masculine standing whereas emotional control or expressing emotion through anger may enhance masculine status [22]. Associated normative attitudes often serve as barriers for men’s proactive (or early) engagement with mental health services [23]. More positively, as gender norms are increasingly questioned, expanded and diversely embodied, there are opportunities for engaging men in mental health promotion. Evidence suggests that some men have begun to challenge, redefine, or incorporate traditional expression of masculinity with health enhancing masculine values [24]. For example, there is evidence that men do not need to engage in all masculine behaviours and that masculine capital may be accrued and used to accommodate some non-masculine or feminine behaviour [25]. Similarly, Emslie et al. found that some men with depression had the resources to construct identities that resist normative masculinities whereas others found it easier to re-interpret potentially feminising experiences as ‘masculine’ [26]. However, to date, most of the available evidence related to men’s mental health has come from clinical populations. It cannot be assumed that general populations will have the same motivations or responsiveness to interventions, compared to men with a diagnosed mental illness. Thus, there is a need to specifically examine non-clinical populations of men, exploring their motivations, experiences, and preferences regarding mental health promotion. The aim of this study was to explore men’s perceptions of mental health and preferences for mental health promotion to inform avenues for men’s mental health promotion.

## Methods

This research was conducted as part of a larger study aimed at developing a gender-sensitised mental health promotion intervention for men living in Sydney, Australia. Focus groups were used to explore men’s perceptions of mental health and preferences for mental health promotion. Focus groups were chosen as a valuable approach to capturing collective group attitudes, norms, and narratives that might not be obtained from individual interviews. Further, as an effective method for engaging men in mental health discourses, focus groups may help to destigmatise mental health by providing a supportive environment, interpersonal support, and validation for men experiencing psychological distress [15,27]. This study was approved by the Human Research Ethics Committee (#ETH18-3184) at the University of Technology Sydney and all participants provided written informed consent prior to participation.

### Recruitment and participants

Participants were men (18+ years) interested in making healthy lifestyle changes, and stakeholders working in men’s health or community-based organisations and groups working to support men’s well-being. Men were recruited using print posters, online media (e.g., Twitter posts, Facebook ads), and information distributed through men’s groups/organisations in the community, with advertisements inviting men to “discuss their motivations, interest, and challenges for staying happy and healthy”. Stakeholder recruitment was facilitated through targeted emails to organisations, support groups, and men specific gatherings and meetings. Organisations were identified that worked directly in men’s health (e.g., men’s health support/advocacy groups) or served and engaged a large proportion of male clientele (i.e., sports clubs). This sampling approach focussed on identifying individuals with knowledge and experience of the research topic. In this study, we used this approach to recruit stakeholders with frontline experience working with men. Our recruitment efforts of stakeholders were expanded using snowball sampling, where participants were encouraged to nominate other individuals with similar critical knowledge and experience that may be interested in participating [28].

### Data collection

A total of 7 focus groups were held between July 2019 and November 2019, including 5 groups of men (6–10 participants per group) and 2 groups of stakeholders (8 per group). Focus groups lasted approximately 2 hours and were facilitated by two members of the research team from non-clinical backgrounds who had prior experience conducting qualitative research with men on topics related to health and well-being. Prior to commencement, men in the study completed a brief sociodemographic survey including age, education, marital/employment status, and household income. Stakeholders did not complete a demographic questionnaire, rather information was recorded regarding their respective organisations and background. These data were collected for descriptive purposes.

The aim of the focus groups was to understand men’s awareness of, interest in, and recommendations for mental health promotion. A consultation-style approach was used, positioning participants as the experts on the topic of men’s thoughts, needs, and interests [29,30]. A semi-structured focus group guide was developed to explore participants’ perceptions of men’s mental health and preferences for mental health promotion, early intervention, and stigma reduction. Open-ended questions were used to stimulate dialogue, promote interaction between participants, and encourage participant self-disclosure without unduly influencing or leading responses. For example, questions included “*What things do men do to help their mental well-being*?” and “*How would it be acceptable to discuss mental health with guys*?” In this way, men in the focus groups were invited to respond to topics based on their personal experiences and perspectives. In responding to similar questions, stakeholder participants were invited to draw on their observations and experiences in engaging men in health promotion initiatives, including those that include mental health components. Using multiple sources of data, often referred to as data triangulation [31], provided the researchers an opportunity to obtain a broader, more comprehensive understanding of the topic under study. Throughout the research process, efforts were made to not unduly influence participants’ responses. Focus groups were audio recorded using a digital recorder Sony IC recorder (ICD-UX560) and transcribed by a research assistant with training and experience in transcribing qualitative research. Completed transcripts were reviewed for accuracy by the lead author prior to analysis.

### Data analysis

We analysed focus group data using inductive thematic analysis, whereby patterns in the data were identified and described to interpret and explain what was said in addressing the research question, “what are men’s perceptions of mental health and preferences for mental health promotion?” The process, as detailed by Braun & Clarke [32] included (1) familiarisation with data, (2) generating initial codes, (3) allocating data segments to the codes, (4) sorting codes to identify potential themes, (5) reviewing themes to determine if they worked in relation to the data, and (6) naming and refining the themes. A coding framework was developed inductively to reflect important ideas represented in the focus groups discussions. For example, initial codes included headings such as *mental health perceptions and practices*, *characteristics and qualities of being a man*, and *gendered social support for mental health*. Two researchers independently coded one transcript to verify the consistency of the framework. Areas of disagreement were discussed, and categories were continually refined, with some codes being subsumed and collapsed while others were expanded to reflect major findings. Coded data were reviewed by the lead author, examining similarities and differences within and across the focus groups to determine preliminary themes.

In the final stage of analysis, we drew upon Connell’s [7] masculinities framework to interpret and unpack the complex gendered dimensions of men’s perceptions and experiences to distil patterns, account for variations in the themes, and refine thematic descriptions. We interpreted gender roles, relations and identities, bridging participants’ narratives to integrate theory and deepen the analyses and advance understandings of men’s mental health and, in turn, inform the development of gender-specific approaches. For example, identifying and integrating complicit, marginalised and subordinate masculinities reflected in the data enabled us to better understand how normative masculinities are embodied, expressed, contested, and (re)negotiated. Representative quotes were chosen that exemplify participants’ perspectives of men’s mental health and preferences for mental health promotion. Study rigor was supported by the use of: a) purposeful sampling, b) strategies to optimise collection of rich and comprehensive data (e.g., data triangulation, using open-ended questions), c) analytical processes to verify and refine hunches and emerging themes (e.g., by comparisons between and within focus groups), and d) representative examples and quotes from the data to support findings. While the findings are not generalizable per se, because participants drew on specific social discourses related to mental health and gender, we believe the findings do hold relevance for some groups beyond the study sample and could inform future research and intervention development.

## Findings

Participants (N = 59) included 43 men and 16 stakeholders. Men had a mean age of 50.65 (SD = 13.75) years and the majority were employed full time (58%), married/domestic partnership (63%), university degree (72%), household income >$100,000 (56%). Participant characteristics are reported in Table 1. Stakeholders worked at commercial health clubs (n = 6), non-profit men’s organisations (n = 5), professional sports organisations (n = 3), and academic institutions (n = 2). Stakeholders self-reported roles included program facilitator, community engagement manager, health/lifestyle coach (e.g., exercise physiologist, personal trainer), motivational speaker, spokesperson/advocate, and researcher. All stakeholders were men.

**Table 1:** Characteristics of men.

	Men (N = 43)
**Age**	
Mean (SD; range: 22–75 years)	50.65 (13.75)
**Highest Level of Education [**n (%)]	
Less than year 12 or equivalent	1 (2)
Year 12 or equivalent	2 (5)
Associate diploma or certificate	9 (31)
University degree	31 (72)
**Marital Status [**n (%)]	
Single/Never married	7 (16)
Married/domestic partnership	27 (63)
Widowed	1 (2)
Divorced	6 (14)
Separated	2 (5)
**Employment Status [**n (%)]	
Full time work	25 (58)
Part time work	11 (26)
Retired	4 (9)
Unemployed	2 (5)
**Household Income [**n (%)]	
Less than $25,000	4 (9)
$25,000 to $34,999	1 (2)
$35,000 to $49,999	1 (2)
$50,000 to $74,999	4 (9)
$75,000 to $99,999	5 (12)
$100,000 to $124,999	7 (16)
More than $125,000	17 (40)
Prefer not to say	3 (7)

Three overarching themes capture men’s perceptions of mental health and preferences for mental health promotion: (1) Roles, identities, and the conceptualisation and concealment of mental health challenges, (2) Constraining social contexts of stigma and gender relations, and (3) Anchoring mental health promotion to acceptable lifestyle practices. These themes are detailed below along with illustrative quotes.

### Roles, identities, and the conceptualisation and concealment of mental health challenges

The interiority (i.e., internalised experiences) of mental health made it challenging for men to conceptualise and express what they were feeling and their level of well-being. For some, it was particularly difficult to know how to act, when circumstances in their lives, such as relocating, divorce, work stress, and shrinking social networks built up and cumulated as unresolved problems. One man described how, retrospectively, he was blinded to his rising stress levels and the flow on impediments to his judgement;

*I’m stressed but it’s death by a thousand cuts. My stress level, I had no idea that my stress level was at that point, and then you’re at the point where you can’t see anything rationally*. *(Men’s Group 1)*

Evident in this quote, and many men’s narratives, were expectations that they could cope with stress (normative masculinity) and the surprise that ‘things’ had built up to the extent that their problem-solving (normative masculinity) was diminished and perhaps disabled.

Participants suggested that challenges for men attending to their mental well-being related to their roles as men, summating that it was, “*the propensity for men to immerse themselves in their work*” *(Men’s Group 1)* and/or men’s “*lack of emotional awareness*” *(Stakeholder Group 1)* that resulted in them neglecting their mental health. In doing so, stakeholders suggested that men’s alignments to masculine norms (e.g., work and stoicism) amplified their risk for mental illness. Men described being on “*automatic pilot*” *(Men’s Group 1)* and marching to the drum of “*1-2-1-2*” *(Men’s Group 4)*, highlighted by this interaction among participants when asked what they do or like to do for their health;

*P1: My health? I work, I look after my family. That is my number 1, number 2 priorities in life*. *Full stop. [General agreement] P2: Work, family. That’s it. (Men’s Group 3)*

Participants discussed how men only considered their health and mental well-being during “*recalibration points*” *(Men’s Group 5)* that required them to reflect and react to crisis situations. One man explained how this occurred when someone (e.g., friend, colleague, family member) or something (e.g., adverse health event, divorce) brought it to their attention;

*It’s a life circumstance or if it’s someone who knows you taps you on the shoulder, ‘take a step out, you need to do this to do this’, that course correction, because I think men don’t find advice, they get consumed, they don’t have those recalibration points, that moment of self-reflection, they go ‘uhhh this is what I want to do in my life’ unless someone taps you on the shoulder or something is forced upon you where you actually have the rude shock*. *(Men’s Group 1)*

Illustrated was discord between routine and purpose, wherein the sense of belonging and purpose that men once drew from keeping busy transactionally obscured the direct pleasures it had once afforded. In essence, the means to an end work ethic became directionless invoking emptiness for some men. Another participant described how these changes could have a dramatic impact on men’s perception of themselves, impacting their mental health; *“It can be quite lonely and isolating when you realise what have you got*. *Men divorce*, *they realise ‘oh mate*, *you’ve got to re-invent*.*’” (Men’s Group 1)* Implicit within these identity threats were reliance on (and relinquishing) idealised masculine roles as the family breadwinner with resounding grief for what had been lost, including the means to an end work ethic defining many men’s lives.

Participants suggested, somewhat provisionally, that perceptions of mental health were changing from a rigid dichotomous perspective of being well or unwell to that of a sliding scale and continuum. One man described his acceptance of this concept;

*In terms of mental health, I think that’s changing. I’m quite comfortable with it. Yes, I do agree I’ve got mental health, ahh [sigh of relief] I’ve got mental health. I think there’s an education piece where mental health is like physical health, it’s the same, you can improve it by using these tools. I think ‘tools’ is a great word, so using mental health tools. Like give me tools and then men will use those tools*. *(Men’s Group 5)*

Reflected in this acceptance of mental health as important, were normative masculinities related to self-reliance and men’s preferences for self-management of their health. Here we also see an example of men’s willingness to invest in actions that can improve mental well-being when they are framed to align with masculine roles and identities (i.e., ‘tools’ for building and constructing). Alternatively, some were resistant, suggesting that mental health was a personal responsibility and that bringing attention to it contributed to the problem. While these comments were often contested, it provided insights to how normative masculinities can be used to silence and censor important topics. For example, one man explained how he believed that discussing mental health may result in a greater prevalence of problems;

*It’s almost asking us to find a problem when the problem should be our responsibility to resolve ourselves. I think that there would be less problems if we didn’t have an advertisement to say ‘hey this is where you go for our problems’, you know? I think it’s promoting not being okay. All of us go through periods where we are not okay and if we weren’t that wouldn’t be life. Before there was Beyond Blue and before there was Lifeline they used to have wristbands with ‘harden the fuck up’ on it. Where if you had a problem, you got more disciplined and you sorted out your problem*. *(Men’s Group 4)*

Here we saw attempts to mute the vulnerabilities being shared by other men by refuting the need for outside help and claiming independence and self-reliance. This assertion provided an example of how dominant masculinities can be expressed and used to contest or defend the marginalisation they can invoke on other men. Participants recognised the challenge of engaging men who are uninterested or unaware of their mental well-being. One man described how he knew that he would benefit but did not perceive himself to be a priority;

*For people like myself, even though intellectually I can embrace this, until I develop a proper sense of self-worth I’m never going to prioritise this. I think it’s really great, but I don’t have the time and that’s because I don’t use my time effectively because I don’t perceive myself as being a priority*. *(Men’s Group 3)*

Articulated here are examples of normative masculinities (work first, selflessness, fatalism) tinged with self-deprecating acknowledgments that he was responsible for being time poor and by extension blinded to introspection.

### Constraining social contexts of stigma and gender relations

Clearly, participants recognised some misalignments between masculine ideals and men’s mental well-being, and it was in this discord that conflict arose between wanting to take preventive action and being constrained by certain social contexts and gender relations. In this space, some men were open to mental health promotion resources and tools for self-management, whereas others remained resistant and attempted to minimise or marginalise vulnerabilities. Men and stakeholders believed that there were considerable barriers and stigmas associated with accessing preventive mental health services. It was clear that men were aware of existing mental health supports and services. Throughout discussions, men highlighted examples of organisations and/or initiatives targeted at mental health, including help-lines (e.g., Lifeline), websites (e.g., Beyond Blue, Black Dog Institute), and mental health campaigns (e.g., R U OK day). Nevertheless, participants believed that most men did not use these services because of a general lack of understanding about these finite resources being for people in crisis. One participant explained;

*I think all the mechanisms that are out there at the moment like your Beyond Blue. Guys are like, ‘I’m struggling, but I’m not to that point.’ We all need to understand that if you are struggling—you are at that point, so kind of identifying that*. *(Men’s Group 1)*

Participants discussed that mental illness (and mental health) remained a largely stigmatised topic, muting potential avenues for discussing or opening up to others–even when they sought out help. This included fears of negative social repercussions including being perceived as inadequate or unmanly. This was especially apparent in workplace settings. One man explained how he sought support from his workplace when he was struggling with his mental health and felt like his concerns were not addressed;

*There is still stigma from workplaces, they preach this ‘R U OK?’ But it’s a bit of a farce*. *I got dragged into HR meetings and in the end they just said, ‘We’ll just sweep it under the carpet, under the chair and hope it all sorts itself out.’ You know? And nothing has been said since. (Men’s Group 3)*

Similarly, one stakeholder recognised that the workplace may not be a safe place to address mental health, and shared this with the men he coached;

*I would coach someone to be very cautious about opening up to too many people, I wouldn’t do it at work. You will pay. There will be programs for you, you might get a flyer [about them]. But [if you open up to others] you could shit your career. I would be coaching them to be careful*. *(Stakeholder Group 1)*

Cautions about raising concerns or asking for help in the workplace were also shared by participants suggesting that this could result in job loss. They advised that men needed to “*curate*” their emotions, as highlighted within this interaction between two men;

P1: *I think in my experience*, *from what I’ve seen*, *there is a lot of lip service that gets paid [to mental health] in an employment situation*…

P2: *I would say that too*!

P1: …*‘Look we are inclusive in our mental health’*, *but when someone puts their hand up and says I’m really struggling*, *everybody goes…*

P2: *You’re sacked*!

P1: …‘*Here’s a number*, *call the helpline’*, *like they are kind of saying*, *‘we didn’t mean it’*. *I guess what I mean is that there is some place to bring some more of yourself and your emotions to the workplace but not too much*. *Curated*. *(Men’s Group 4)*

Seen here were stigmas assigned to mental illness to the point that disclosures of vulnerabilities were incompatible with paid work, raising issues about the discord between what employers say and do with regards to promoting men’s mental health. Some men faced similar challenges with negotiating their participation in social or support groups for men. In general, men highlighted that they did not always feel comfortable sharing or discussing their thoughts and feelings with others. In doing so, we see how masculinities are not self-reproducing and require considerable effort to maintain within social contexts. Even in close social circles, gender roles can be policed and contested. One man described how opening up about mental health was often seen as a sign of weakness and resulted in being ridiculed and ostracised by peers. He recounted, “*I told a mate that I was coming [to a men’s group] and he laughed and I haven’t spoken to him since*.” *(Men’s Group 3)* Publicly acknowledging or discussing their involvement in a men’s group with their friends, family, or colleagues was also viewed as risky. One man examined how men’s groups were portrayed in the media as hosting marginalised, subordinate ‘less-than’ men, and how his own participation in a men’s group was goaded by his daughter;

*…one of his [the actor] friends in the show says he’s going to a men’s group and he [the actor] just absolutely takes the piss out of him*. *It’s the way he does it too in this very sarcastic way and it’s left at that*. *It doesn’t feel safe for me to tell most people that I even go to a men’s group*. *My daughter jokingly*, *who is 19*, *calls it your ‘crying group’*. *And I mean*, *how emasculating can you get*? *That is really emasculating*.” *(Men’s Group 3)*

Revealed here was the co-construction of masculinities and the shaming that can be assigned for transgressing ideals of self-reliance and stoicism. Also implicit were views that men who wanted (and drew benefit from) the company of other men to connect on a deeper level were suspect and subordinate. Identifying the gathering as a ‘crying group’ signals marginalised masculinity, recognised by the participant and referencing fear for men expressing what is assigned and asserted as unregulated feminine emotions.

### Anchoring mental health promotion to acceptable lifestyle practices

Participants had ideas about how community-based mental health promotion could be offered to men in acceptable ways. The concept of “*anchoring*” was proposed, whereby mental health promotion could be accomplished by circumventing stigmas or embedding behaviours within avenues that supported men’s mental health. Considering the challenges with engaging men in community-based mental health promotion, participants proposed strategies for discussion, education, and management of one’s mental health.

Participants discussed the importance of having mental health promotion anchored in peer social settings and described that the major draw of this approach was “*doing things together for the benefit of each other*” *(Men’s Group 2)*. One stakeholder explained;

*In getting help*, *I am helping others*. *Maybe men aren’t as good at getting help as they should be*, *but blokes really like giving help*, *offering help*, *being useful*. *So you train people to look after their mates rather than train them to look out for themselves*, *but in the process they learn how to look out for themselves*. *(Stakeholder’s Group 2)*

In this sense, comradery, teamwork, and doing health for others highlighted health-related masculine values of selflessness and openness. Men explained how this provided opportunities for conversation to occur naturally;

*I found that places like Men’s Sheds, where it’s never overt, there’s a lot of debriefing, there’s a lot of talking about feelings, although they wouldn’t admit to that*. *But it’s that sort of safety that men feel around other men often helps with their mental health stuff a lot. (Men’s Group 2)*

A key challenge remained with regards to engaging those men that were uninterested or unaware of promoting their mental health. Participants suggested that targeting groups of men with common interests may provide an entry point to start conversations around mental health promotion. One man explored this, speculating that champions within these groups could be used to initiate conversations;

*So how do you get through to guys that don’t even know they have an issue? I think that is quite challenging. How do you infiltrate that or get one person that is the influencer of that group to start to go, ‘Over here these guys are having this conversation*, *we haven’t had that one!’ (Men’s Group 5)*

Similarly, one man referenced the need for a range of tailored mental health supports for diverse subgroups of men with the caveat that there was a need to be very careful when broaching the topic with some subgroups;

*I work with Aboriginal populations but even in the mainstream there’s a growing awareness of the need for addressing mental health needs.…Depending on the demographic you are trying to approach, that might turn some people off. I think it’s how you promote it, how you use your words within whatever promotional information materials you have. Say for our population group, mental health is a massive need to address anything from mild anxiety and social/emotional well-being to full blown depression and intergenerational trauma*. *(Men’s Group 2)*

Implicated here is not only the need to recognise the diverse backgrounds of men but also the diversity of masculinities both within and across subgroups of men that need to be considered in designing tailored approaches for promoting men’s mental health.

Participants provided examples of activities that draw men together and that offer a context for integrating mental health promotion including sport (e.g., rugby, football), hobbies (e.g., gardening, woodworking, car mechanics), or other interests (e.g., mountain biking, surfing). Many described how the perceived enjoyment of these activities would be primary motivator to participate. One stakeholder shared his observations that under the right circumstances, men were open to engaging in discussion;

*People say men don’t talk*, *well that’s bullshit*, *once they’re in the right space you can’t shut them up*. *And that to me is the point*, *how do we get them there and fitness and health could be that*. *And then you could tie the mental health into this*. *The reason can’t be come and talk about mental health and have a bit of exercise*. *It’s got to be the other way around in my view*.” *(Stakeholder Group 2)*

To avoid overt discussions about mental health with men, another suggested that this information should be embedded, or anchored, around other behaviours.

*Maybe it needs to be focused on the health aspect and not the mental side. I guess if it was a group that was centred around—let’s come in and improve our health as opposed to let’s come in and improve our mental health—that might just be a stigma just on the one word included in the sentence. So how it’s written and how it’s communicated will be imperative to how the engagement occurs*. *(Stakeholder Group 2)*

In this context, discussions could take place that would not have otherwise occurred. One man provided an example of how exercise provided an avenue to allow men to have emotive discussions;

*I find a lot of my mates get out running, it’s the only time I ever get them vulnerable. I think it’s because I’m not looking at them, they’re looking straight ahead. You know you have D&Ms [deep and meaningful conversations] with your mates in the car because you’re sitting there facing forwards! It’s really interesting I think that’s the only way that a lot of men will start to get comfortable having vulnerable conversations. It’s exercise and not being face to face*. *(Men’s Group 5)*

Within these settings, participants also suggested that there were opportunities to incorporate education and information regarding mental health promotion. The example of **“***what is going on in your mind without labelling it*” *(Men’s Group 2)* and using a “*multidimensional approach rather than sitting around talking*” *(Men’s Group 1)* was used to describe opportunities for education. For example, there was agreement that following a workout or exercise session, it would be acceptable to engage men in a guided mindfulness activity as a post-workout cooldown.

## Discussion

Men’s mental health promotion interventions are emergent and there is a need for evaluative evidence to identify strategies that enable mental health promotion and early intervention gains in community-based settings [15,33]. We conducted focus groups with men and stakeholders in Sydney, Australia, to explore men’s perceptions of mental health and preferences for mental health promotion to inform avenues for men’s mental health promotion. Our study findings contribute important insights into the complex relationship between men’s mental health and masculinities and may help to inform optimal approaches to men’s mental health promotion. Participants highlighted that mental health challenges were often hard to perceive due to perceptions of men’s restrictive emotionality and emotional awareness. Further, participants revealed how social context and the policing of gender roles often obscured opportunities for discussing mental health and help-seeking behaviour. Finally, participants identified strategies to men’s mental health promotion including opportunities to leverage men’s social practices related to reciprocity, normalising mental health promotion relative to other behaviours, and embedding mental health promotion within acceptable lifestyle practices. Elucidated within these findings are directions for mental health promotion within community-based settings.

### Men’s mental health promotion

Reflected in the focus group discussions was a shared understanding of the barriers to mental health promotion relating to perceptions of men’s restrictive emotionality and emotional awareness, the conceptualisation and interiority of mental health, and stigmatisation of mental health promotion and practices. However, as noted by Emslie et al. [34], the relationship between gender and mental health is not straightforward and both men and women may find it difficult to recognise and articulate mental health problems. Nonetheless, notable throughout the present findings was the influence of normative masculinities including fatalism and self-reliance and examples of how counter masculinities (e.g., vulnerability) could be contested and muted. Of course, cultures and gender norms shift, and men emphasised the need to connect with other men to strategize the sharing and solving of their collective mental health challenges. It is the role of advocates, researchers, and policymakers to collaborate to facilitate these shifts and garner opportunities for healthful actions and discussions within existing and progressive paradigms. Further, future gender comparative work should be extended to breaking down gender binaries by inclusively examining transgender and gender diverse people to explore how gender ideologies influence mental health to inform tailored approaches to mental health promotion.

As the challenges raised by participants predominantly centred on social factors (e.g., stigmatisation), as distinct from other determinants of health (e.g., biological), it stands to reason that the potential remedies may also originate from the gendered places that invoked the challenges. For example, the workplace was identified as a space where structural and institutional gender silenced men suspected of mental illness and/or marginalised those who revealed they had mental health challenges. Highlighted here was the lack of psychological safety and perception that acknowledging mental health problems risked ridicule, judgement, or recourse within this context. Participants suggested men could be engaged in mental health promotion by leveraging men’s social practices related to reciprocity, normalising mental health promotion relative to other behaviours, and embedding mental health promotion within acceptable lifestyle practices. Clearly, some of the nuance to engaging men lies within the framing of discussions and actions that can be argued as therapeutic without compromising masculine capital [35]. Highlighted here are examples of how masculinities may be negotiated and expanded to encompass more mental health-related masculine values. For example, taking action for the benefit of others, as well as oneself, demonstrates health-related masculine values of selflessness and openness serves to engage and reflect normative masculinities of strength and self-reliance [36].

Paradoxically, campaigns and organisations directly marketing “mental health” are unlikely to attract men who are resistant to engaging help due to their alignment to masculine ideals that triage out or entirely deny *their* mental health challenges. While these campaigns and organisations should be applauded for their work to raise awareness and provide mental health supports, many men did not believe that their challenges fell within the scope of these formal services; instead suggesting alternative approaches to mental health promotion (e.g., connecting with and supporting others, anchoring conversations and skill building opportunities in activities that engage men). While efforts should be made to clarify available support, these organisations face inherent challenges that, by overtly naming mental health, many men will be reticent to engage for fear of being perceived, seen and judged as mentally unwell [2,5]. As such, there is clear scope to target mental health promotion from the perspective of prevention. Important, however, will be anchoring approaches in community contexts where men live and work, that allow for mutual help garnered by connecting with other men who have and manage similar or relatable challenges. Here the value of anchoring mental health shines as a strategy for engaging those that would otherwise not present to mental health programs and services.

### Anchoring mental health promotion to existing communities

Mental health promotion provides a unique opportunity to prevent the onset, delay recurrence, and decrease the impact of a range of mental health disorders. In line with Oliffe et al. [16], community-based programs can be familiar and acceptable for men. Embedding mental health promotion within existing communities is especially important as it provides a safe space to talk about issues of concern whilst engaging in social activities with individuals who share common interests, motivations, and/or qualities [13]. Key considerations include the literacy and language used by men (and reflected in programs), the need for program pacing to blend activities and talk, and matching the environment to end-user sub-groups. It was clear that men wanted opportunities to talk and, while activities were important, they craved depth of discussion. The intersections of culture and gender also highlighted place-based considerations recognising normative masculinities as geographically anchored. By extension, men aligned to, and argued against dominant masculine ideals embodying an array of configurations and contexts in those milieus.

The importance of men working ‘shoulder-to-shoulder’ in an environment that combines purposeful social activity with opportunities to debrief cannot be overstated. Participating in traditionally masculine activities provides participants permission to express themselves openly, facilitating companionship and closeness [37]. Here we find evidence for the importance of this social infrastructure, scaffolded by men’s common interests and hobbies, which provides the health enhancing contexts to stimulate mental health and well-being. One noteworthy program that has been successful at engaging older men in psychosocial support is the Men’s Sheds movement [37].We find support for this approach with young and middle-aged men suggesting a myriad of social settings that may be leveraged to engage men in mental health promotion (e.g., sport, hobbies, clubs, social settings). While the demand is clearly there for men’s programs, gender-sensitive designs and evaluations are key [38]. Recommended in this regard are concerted consistent efforts for building community-based men’s mental health programs as a means to guide the future work of others, and ideally make possible sustainable and scaled programs.

### Anchoring mental health promotion to lifestyle interventions

Lifestyle interventions provide another opportunity to target men’s mental health promotion. Interest in developing gender-sensitised interventions that engage and retain men have been successful at promoting weight loss, healthy eating, and physical activity [39–41]. To date, the large majority of lifestyle interventions have only targeted mental health as an adjunct outcome to changes in physical activity and/or diet. A recent systemic review by Drew et al. [42] revealed that lifestyle interventions demonstrated potential for improvements to men’s mental health-related quality of life, self-esteem, and positive affect. However, none of the included studies targeted mental health as a primary outcome. Further, the authors noted that very few studies included any direct mental health support within the lifestyle intervention, relying on the benefits of increased physical activity and improved diet to influence participants mental health outcomes [42].

Our findings point directly to opportunities within these lifestyle programs to bundle mental health promotion with other health promoting behaviours. Within these settings, it is possible to take full advantage of men’s openness to new information, experiences and skill-building related to health-enhancing behaviours to introduce strategies for mental well-being. For example, following a 12-week lifestyle intervention, participants reported that, as the program progressed, they began to consider healthy changes and behaviours that they would not have previously considered to be acceptable [43]. By coupling mental health promotion to other lifestyle changes, tips and tools for promoting mental well-being can be enveloped with physical activity and healthy eating in ways to integrate and evaluate men’s mental well-being. Here participants’ suggestions regarding tools and tactics can afford men autonomy in selecting *their* strategies for advancing mental health. This performativity and self-reliance, as a normative masculine frame, can also destigmatise by avoiding treatment labels and the implicit indebtedness of *receiving* professional help. In this regard, tools and tactics demand action, involvement, and skill–and made available through lobbying men to take up these challenges are the visibility of a hard work-ethic and investment in effective self-management. Notwithstanding the benefits of integrating mental health into lifestyle programs, supportive environments are also needed to enable and support men’s mental health. In this regard, although workplace health promotion programs tailored for men demonstrate health behaviour changes confirming work as an important setting for inserting mental health promotion [44,45], formal policies and practices are key to norming conversations and help-seeking among working men.

## Strengths and limitations

These findings should be considered in light of the strengths and limitations of this research. Notably, the present study was strengthened by the relatively large sample of participants, including both men and stakeholders working in men’s health, representing diverse backgrounds, experiences and interests. As the sample of men were invited on the pretense of discussing overall health, a broader array of perspectives regarding mental health may have been captured. Limitations notwithstanding, the collective nature of focus groups may result in some participant voices not being heard, including those participants who may have been reticent to disclose personal information to the group. Being aware of these issues, the researchers attempted to be as inclusive and open as possible, establishing a comfortable discussion environment and giving all participants a voice during discussions.

The current study cohort was drawn from a single urban location which may mean the findings are contextually tied to the specific locale and not necessarily reflective of men in other regions, including rural and remote locations where traditional masculine norms may be upheld with greater resolve. Additionally, the sample was comprised mainly of University educated men (73%) with a moderate-to-high level of income, thus in future it is important to include men from less educated/affluent and socially diverse groups. Moreover, it is important to incorporate men from other ethnic/cultural backgrounds both within the Australian context (e.g., Aboriginal and Torres Strait Islander men) and beyond. Overall, the current (de)limitations provide some direction for future research which might usefully include larger longitudinal and mixed methods studies to distil with greater certainty the similarities and differences for men’s health practices in these contexts.

## Conclusion

The findings from the current study demonstrate the need to provide better direction for gender-sensitised approaches to community-based mental health promotion for men. Especially evident was the influence of gender roles, relations and identities as well as the importance of framing conversations in ways that resonate with potential end users. Clearly strategies are needed that address, or at least consider, men’s restrictive emotionality and emotional awareness, the interiority of mental health, and stigmatisation of mental health promotion and practices. Highlighted throughout, from both men and stakeholders, was the significance of advancing men’s mental health and wellbeing by anchoring mental health promotion to acceptable locations, contexts and behaviours. In doing so, programs and services may begin to align with gender-specific and -transformative approaches by considering men’s specific needs and addressing harmful gender norms, roles and relations [46]. Accordingly, the effectiveness of potential remedies is deeply reliant on knowing, engaging, and working with–as well as reworking–some masculine ideals about how men engage in mental health promotion in specific locales.
